# Fs-Laser Fabricated Miniature Fabry–Perot Interferometer in a No-Core Fiber for High-Temperature Applications †

**DOI:** 10.3390/s23187754

**Published:** 2023-09-08

**Authors:** Chen Zhu, Osamah Alsalman, Jie Huang

**Affiliations:** 1 Research Center for Optical Fiber Sensing, Zhejiang Laboratory, Hangzhou 311100, China; 2 Department of Electrical Engineering, College of Engineering, King Saud University, P.O. Box 800, Riyadh 11421, Saudi Arabia; 3 Department of Electrical and Computer Engineering, Missouri University of Science and Technology, Rolla, MO 65409, USA

**Keywords:** Fabry–Perot interferometer, femtosecond laser fabrication, high temperature, fiber optic sensor

## Abstract

This paper reports a fiber in-line Fabry–Perot interferometer (FPI) fabricated in a no-core fiber using the direct femtosecond laser writing technique for high-temperature sensing applications. Two in-line reflectors are directly inscribed in a no-core fiber to construct a low-finesse FPI. Fringe visibility greater than 10 dB is obtained from the reflection spectra of the fabricated no-core fiber FPIs. Temperature responses of a prototype no-core fiber FPI are characterized up to 1000 °C. The proposed configuration is compact and easy to fabricate, making it attractive for sensing applications in high-temperature harsh environments.

## 1. Introduction

Among various types of fiber-optic interferometers, fiber in-line Fabry–Perot interferometers (FPIs) offer unique features, such as compactness, linear response, ease of signal demodulation, and ease of fabrication [1]. A typical FPI consists of two reflectors with either a solid cavity (e.g., a section of optical fiber as the cavity medium) or a hollow cavity (e.g., air as the cavity medium). The light reflections from the two reflectors superimpose to generate an interference pattern, through which the optical path difference (OPD) of the FPI can be determined. Any parameter that can be correlated to the OPD of an FPI can be measured [2], such as strain [3], temperature [4], refractive index [5], pressure [6], displacement [7,8], etc.

Different techniques have been explored to create the two reflectors in an optical fiber to construct an FPI. Among them, direct femtosecond- (fs-) laser inscription has been demonstrated as a unique and advantageous method that is unsurpassable by other types of techniques. When exposed to tightly focused fs-laser pulses, the refractive index of an optical fiber core is modified permanently [9,10]. The impedance mismatch between the unmodified region and the fs-laser-modified region results in a reflection of the transmitting light. The reflectivity of the created reflector can be tuned by adjusting the parameters of the fs-laser pulses. Using the fs-laser micromachining technique, a series of assembly-free and ultra-compact FPIs have been demonstrated. Importantly, the reflectors fabricated by a femtosecond laser are stable at elevated temperatures, making these FPIs attractive in high-temperature applications [4,6].

Single FPIs [4,5,6,11,12,13], cascaded FPIs [14,15], and parallel structured FPIs [16] were successfully fabricated in single-mode fibers (SMFs) via direct fs-laser inscription. Most of these compact fiber-inline sensor devices were demonstrated for high-temperature applications. However, an intrinsic limitation of these sensor devices is that the sensing platform, i.e., the SMF, is made with germanium-doped silica, which would suffer from dopant diffusing in the optical fiber core at elevated temperatures (>800 °C) [17]. The diffusion of the dopant results in the spectrum drift of the sensor, thus providing misleading information about measurands. Therefore, to address this issue, dopant-free optical fibers, such as the no-core fiber (NCF, e.g., fused silica fiber, single-crystal fiber), need to be explored for sensing applications in extremely high-temperature environments [18,19,20,21].

In this paper, we present a miniature in-line FPI in an NCF, for the first time, for high-temperature applications. The FPI is constructed using two fiber in-line reflectors fabricated with an fs-laser. Interference patterns with fringe visibility greater than 10 dB are obtained from the fabricated NCF-FPIs. The temperature responses of a prototype NCF-FPI are characterized up to 1000 °C. Strain responses are also demonstrated.

## 2. Sensor Fabrication and Principle

An fs-laser system (Spirit One, Spectra-Physics, MKS Instrument, Andover, MN, USA) producing laser pulses with a width < 400 fs at a repetition rate of 200 kHz and with a central wavelength of 1040 nm was employed in the fabrication. The integrated high-efficiency second harmonic generation module enables the central wavelength of the fs-laser amplifier to switch between 1040 nm and 520 nm. The home-built micromachining system is similar to the setup in [22], including an fs-laser amplifier and a versatile manufacturing workstation (femtoFBG, Newport Corporation, Irvine, CA, USA). The optical fiber was immersed in index-matching gel and sandwiched between two glass slides. The optical fiber was secured onto a three-dimensional translation stage assembly which offers a displacement resolution of 0.05 μm along the horizontal direction and a displacement resolution of <1 nm along the vertical direction. The tilt and rotation of the optical fiber secured onto the stage could also be adjusted. A microscope objective (ZEISS 40X) with a numerical aperture of 0.75 was used to focus the laser beam into the desired position in the optical fiber. The average laser power delivered to the optical fiber could be varied using a half-wave plate and a Glan-laser polarizer, both of which were included in the workstation. An online monitoring system composed of a CMOS camera and lens assembly was used to assist with the alignment and visualization of the fabrication process.

**Figure 1:**
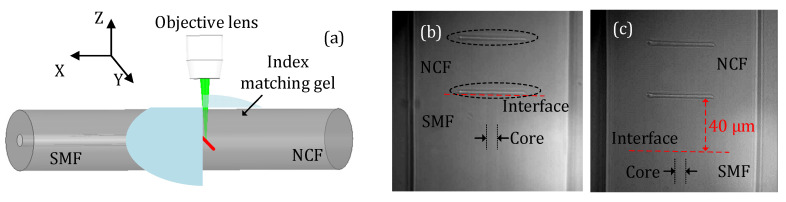
Illustration of the device fabrication and microscope images of the fabricated device samples. (**a**) Schematic of the fabrication process. (**b**) Microscope image of a device sample where the first reflector (the lower one) was very close (~2 μm) to the fusion splicing interface between the SMF and NCF. (**c**) Microscope image of a device sample where the first reflector was 40 μm above the interface.

A section of NCF (FG125LA, THORLABS, Newton, MA, USA) made of silica was first connected to an SMF (SMF-28, Corning, Corning, NY, USA) using a fusion splicer (Fujikura 70s, Japan). The diameter of the NCF was 125 μm, which matched well with that of a standard SMF. The hybrid fiber structure (i.e., SMF-NCF) was then secured to the workstation for fs-laser irradiation. Figure 1 includes a schematic diagram of the fabrication process and microscope images of the fabricated device samples. In the fabrication, the optical fiber was translated along the Y-direction (as indicated in Figure 1a) at a constant velocity of 10 μm/s. The repetition rate of the laser was reduced to 5 kHz by means of the laser internal pulse picker. The energy of a single laser pulse delivered to the optical fiber was approximately 600 nJ. As can be seen in Figure 1b, two reflectors (marked in the dashed oval) were fabricated in the NCF with a distance of ~40 μm. In this way, an FPI was constructed. The lengths of the two reflectors were ~50 μm. The first reflector (the lower one) was very close to the fusion splicing interface between the SMF and the NCF, i.e., a 2 μm offset. Figure 1c presents a microscope image of another device sample where the first reflector was ~40 μm above the fusion splicing interface, i.e., a 40 μm offset. Owing to the high transmission loss of the NCF, we expect that the device sample shown in Figure 1b would have a better signal quality than the device sample shown in Figure 1c. Note that the free end of the NCF was broken to avoid additional reflection.

The reflection spectrum of an NCF-FPI can be approximated as [1]:

(1) $$I=I_{1}+I_{2}+2\sqrt{I_{1}I_{2}}\cos(\frac{4\pi nL}{\lambda }+\phi )$$

where *I*_1_ and *I*_2_ represent the reflected light intensities from the first reflector and the second reflector, respectively; *n* is the refractive index of the NCF; *L* is the cavity length of the FPI; *ϕ* is the phase term of the interferometer; and *λ* is the wavelength. According to the phase-matching condition, the resonance wavelength of the NCF-FPI can be expressed as:

(2) $$\lambda_{res}=\frac{4nL}{2m+1}$$

where *m* is a non-negative integer, denoting the resonance order. The free spectral range (*FSR*) is then given by:

(3) $$FSR=\frac{\lambda^{2}}{2nL}$$

Therefore, assuming the refractive index of the NCF and the cavity length of the FPI are 1.444 and 40 μm, the theoretical *FSR* can be calculated to be ~21 nm for an interrogation wavelength of 1550 nm.

**Figure 2:**
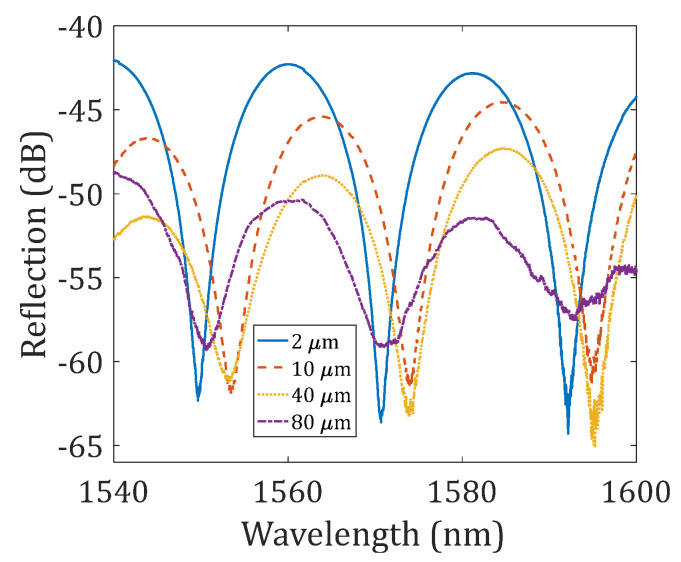
Reflection spectra of the NCF-FPIs. These four device samples have different offset distances between the first reflector and the fusion splicing interface.

The fabricated device samples were characterized using a broadband source (Thorlabs ASE-FL7002-C4, 1530–1610 nm) and an optical spectrum analyzer (ANDO AQ6317B, Japan). Figure 2 presents the measured reflection spectra of several device samples; the difference between these device samples is the offset distance of the first reflector to the fusion splicing interface, i.e., 2 μm (shown in Figure 1b), 10 μm, 40 μm (shown in Figure 1c), and 80 μm. The *FSR* for all the reflection spectra shown in Figure 2 was found to be approximately 21 nm, which matched well with the theoretical value. For the 2 μm-offset device sample, the fringe visibility of the interference pattern was found to be >20 dB; as the offset distance increased to 10 μm, 40 μm, and 80 μm, the fringe visibility decreased to ~15 dB, ~13 dB, and ~10 dB, respectively. The total reflectivity of the FPI also decreased as the offset distance increased. A modulation envelope could also be observed in the reflection spectra, which was due to the additional reflections that occurred at the two boundaries of the first reflector (the reflector has a thickness of ~2.5 μm).

## 3. Results

**Figure 3:**
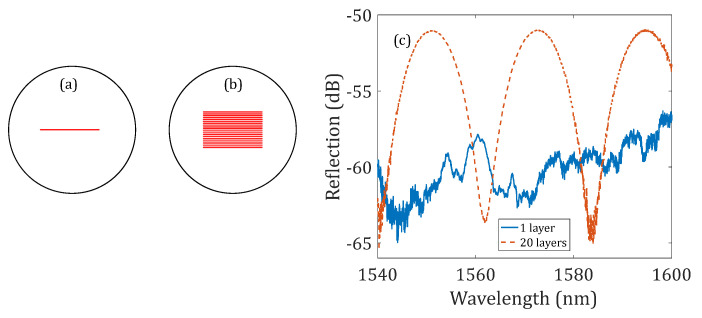
Demonstration of using multi-layer line reflectors for reflectivity enhancement. Illustration of the (**a**) 1-layer line reflector and (**b**) 20-layer line reflector. (**c**) Reflection spectra of the NCF-FPIs with two 1-layer line reflectors and two 20-layer line reflectors. The offset distance of the first reflector in the two NCF-FPIs to the fusion splicing interface was 120 μm.

Figure 3 shows the characterization of the other two device samples, where the offset distances of the first reflector to the fusion splicing interface were increased to 120 μm. The two reflectors of the first device sample were fabricated by inscribing a single line along the Y-direction, as illustrated in Figure 3a (the same method used to fabricate the devices shown in Figure 2); the reflectors of the second device sample were fabricated by translating the stage (i.e., the optical fiber) along the Y- and Z-directions so that multiple layers of the line reflector were fabricated, as schematically illustrated in Figure 3b. The separation between each layer was 1 μm. Figure 3c shows the measured reflection spectra of these two device samples with 1-layer and 20-layer line reflectors. As the offset of the first reflector to the fusion splicing interface increased to 120 μm, no interference pattern was obtained from the NCF-FPI with two 1-layer line reflectors due to the high transmission loss of the NCF. However, the reflectivity of the NCF-FPI with two 20-layer line reflectors was greatly increased; a good interference pattern was also obtained from the device sample with 20-layer line reflectors with fringe visibility greater than 10 dB. Therefore, this result demonstrates that by increasing the effective area of the fiber in-line reflector, the reflectivity can be enhanced, thereby resulting in a higher-quality interference pattern that can be used for sensing applications.

**Figure 4:**
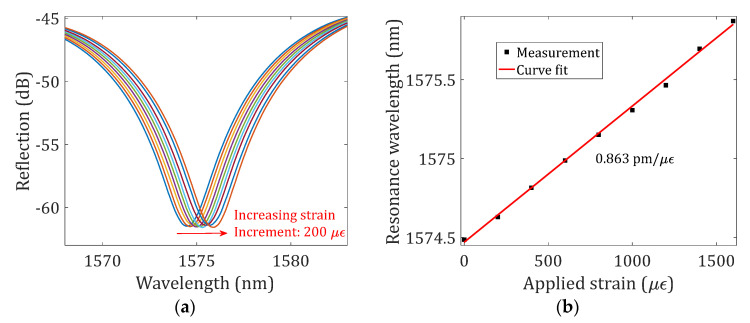
Strain responses of the NCF-FPI. (**a**) Evolution of the reflection spectrum for different settings of applied tensile strain. (**b**) The measured dip wavelengths as a function of applied strains. A linear curve fit was applied to the measured dataset, and the determined strain sensitivity is indicated in the figure.

To verify their functionality, the strain responses of the NCF-FPI (the device sample with a 10 μm offset and two 1-layer line reflectors as given in Figure 2) were first characterized at room temperature. The sensor device was centered between two translation stages (OMTOOLS, HFA-XYZ, Wuhan, China) fixed on an optical table with a separation distance of 150 mm. One stage was forced outwards to elongate the secured optical fiber section in increments of 30 μm, thus applying strains in increments of 200 microstrains (με) to the sensor device along the axial direction. Figure 4 presents the investigation results. The evolution of the reflection spectrum at around 1575 nm for different settings of applied strain is shown in Figure 4a. A low-pass filter was applied to the recorded spectra. The reflection spectrum shifted to the long-wavelength region as the applied strain increased, as can be expected based on Equation (2). Figure 4b shows the dip wavelength as a function of the applied tensile strain. A linear curve fit was applied to the measured datasets; the slope, i.e., the strain measurement sensitivity, was determined to be 0.863 pm/με with an R-squared of 0.9978.

**Figure 5:**
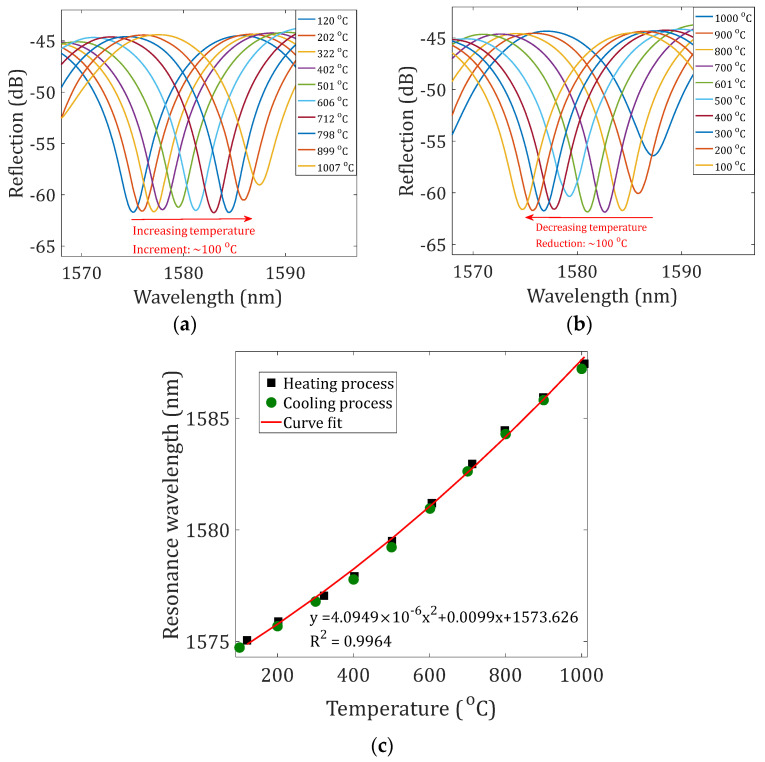
Temperature responses of the NCF-FPI. Evolution of the reflection spectrum for different temperature settings in the (**a**) heating process and (**b**) cooling process. (**c**) The measured resonance wavelengths in the heating and cooling processes as functions of applied temperatures. A second-order polynomial curve fit was applied to the measured data points.

The temperature responses of the NCF-FPI (the device sample with 10 μm offset and 1-layer reflectors) were tested by placing the sensor device in a tubular furnace (Lindberg BLUE M, Thermal SCIENTIFIC, Waltham, MA, USA). The temperature in the furnace was increased from 100 to 1000 °C in increments of 100 °C and subsequently decreased to 100 °C with an average ramp rate of ~20 °C/min. The recorded reflection spectra of the sensor for different temperature settings in the processes of increasing and decreasing temperature are plotted in Figure 5a,b, respectively. The spectra were filtered using a low-pass filter. The spectrum shifted to the long-wavelength region as the temperature increased and recovered as the temperature decreased. Figure 5c shows the measured resonance wavelengths as a function of temperatures during the heating and cooling processes. The responses of the sensor in the heating processes and cooling processes matched well. A second-order polynomial curve fit was applied to the measured datasets; the fitting results are included in Figure 5c. Alternatively, by dividing the temperature range into two separate regions, i.e., 100–400 °C and 400–1000 °C, linear curve fits can also be applied. The slopes at 100–400 °C and 400–1000 °C were determined to be 9.865 pm/°C and 15.95 pm/°C with R-squared of 0.9993 and 0.9988, respectively. The obtained temperature sensitivities of the NCF-FPI matched well with previously reported values of SMF-FPIs [13,16,23].

## 4. Conclusions

In conclusion, we report miniature fiber in-line FPIs fabricated in no-core fibers for high-temperature applications. The sensor device was fabricated using the direct femtosecond laser inscription technique. Interference patterns with fringe visibility greater than 10 dB were obtained for several device samples. The strain responses of a prototype device were characterized. Temperature measurements up to 1000 °C were demonstrated, showing measurement sensitivity of 9.865 pm/°C and 15.95 pm/°C in the temperature range of 100–400 °C and 400–1000 °C, respectively. The proposed sensing structure holds several advantages, such as ease of fabrication, small size, and high-temperature survivability. It is expected that the proposed sensing configuration can be implemented onto NCFs made of other materials (e.g., sapphire single-crystal fibers) to further enhance the high temperature tolerance of the device in extremely high-temperature harsh environments.

## Data Availability

The data presented in this study are available on request from the corresponding author.
